# Publications of Massachusetts Homœopathic Medical Society, 1887

**Published:** 1888-07

**Authors:** 


PUBLICATIONS OF THE MASSACHUSETTS HOMŒOPATHIC MEDICAL SOCIETY, 1887.

This very neat volume of some two hundred and forty pages gives the proceedings and the papers read at the annual and semi-annual meeting of the Massachusetts Homœopathic Society for 1887. It contains some good papers and some not quite so good, homœopathically considered.

